# De Ritis Ratio as a Prognostic Marker for Mortality in Moderate-to-Severe Traumatic Brain Injury: A Propensity Score-Matched Analysis

**DOI:** 10.3390/diagnostics15192416

**Published:** 2025-09-23

**Authors:** You-Cheng Lin, Ching-Hua Tsai, Wei-Ti Su, Shiun-Yuan Hsu, Ching-Hua Hsieh, Cen-Hung Lin

**Affiliations:** 1 Department of Neurosurgery, Kaohsiung Chang Gung Memorial Hospital, Chang Gung University and College of Medicine, Kaohsiung 83301, Taiwan; 2 Department of Trauma Surgery, Kaohsiung Chang Gung Memorial Hospital, Chang Gung University and College of Medicine, Kaohsiung 83301, Taiwan; 3 Department of Plastic Surgery, Kaohsiung Chang Gung Memorial Hospital, Chang Gung University and College of Medicine, Kaohsiung 83301, Taiwan; 4 Department of Plastic Surgery, Kaohsiung Municipal Ta-Tung Hospital, Kaohsiung 80145, Taiwan

**Keywords:** traumatic brain injury, De Ritis ratio, mortality, prognostic marker, head injury

## Abstract

**Background:** Traumatic brain injury (TBI) remains a significant cause of global mortality and morbidity. This study investigated the association between initial De Ritis ratio (serum AST/ALT) and mortality in moderate-to-severe TBI patients. **Methods:** This retrospective study analyzed 4415 adult patients with moderate-to-severe TBI (head/neck AIS ≥ 3) from January 2009 to December 2020. Patients were categorized into three groups based on De Ritis ratios: ≤1.2 (*n* = 1296), 1.2 < ratio ≤ 1.64 (*n* = 1499), and >1.64 (*n* = 1620). Propensity score matching was performed to adjust for baseline characteristics including age, sex, comorbidities, Glasgow Coma Scale, and Injury Severity Score. **Results:** Higher De Ritis ratios were also associated with increased intensive care unit admission rates and longer hospital stays. After propensity score matching, patients with De Ritis ratio > 1.64 showed significantly higher mortality rates compared to the control group (10.3% vs. 7.9%, odds ratio (OR) = 1.33, 95% confidence interval (CI) 1.01–1.77, *p* = 0.046). No significant mortality difference was found between patients with De Ritis ratio ≤1.2 and the control group. **Conclusions:** Initial De Ritis ratio>1.64 serves as an independent predictor of mortality in moderate-to-severe TBI patients. This readily available laboratory parameter could provide valuable prognostic information for early risk stratification in TBI management.

## 1. Introduction

Traumatic brain injury (TBI) represents a significant global health burden, affecting millions of individuals annually across all age groups and contributing substantially to worldwide morbidity and mortality rates [1]. TBI occurs when sudden external forces impact the head, disrupting normal brain function through a complex pathophysiological process that unfolds in two distinct phases [2]. The primary phase involves immediate mechanical damage, resulting in direct brain tissue injury and neural cell necrosis. This is followed by a secondary phase, which develops over minutes to hours, characterized by the activation of complex cellular cascades leading to further necrosis and apoptosis. During this secondary phase, inflammatory responses compromise the blood-brain barrier, exacerbating cerebral edema [3]. In severe cases, acute TBI can trigger systemic inflammatory response syndrome, potentially progressing to multi-organ dysfunction syndrome [4]. Given this complex pathophysiology, TBI outcomes are determined by multiple factors, including the severity of both primary and secondary injuries, pre-existing comorbidities, and the effectiveness of therapeutic interventions.

Several studies reveal that TBI induces significant hematological changes, reflected in blood-drawn laboratory data, which can serve as biomarkers for severity and prognosis. TBI patients consistently show elevated levels of inflammatory cytokines such as IL-6, IL-8, and TNF, which correlate with injury severity and poorer outcomes [5]. Hematologic profiles often reveal reduced hemoglobin and hematocrit levels shortly after injury, while white blood cell counts spike, indicating acute inflammatory responses [6]. Coagulation abnormalities, such as increased D-dimer and fibrin degradation products, reflect the risk of intracranial hemorrhage and thrombosis [7]. Renal function can also be impaired, as indicated by increased creatinine and blood urea nitrogen levels, suggesting that TBI-induced hemodynamic instability or secondary injury processes affect kidney function [7]. Liver function tests often show elevations in aspartate aminotransferase (AST) and alanine aminotransferase (ALT), reflecting hepatic stress or damage, which may be exacerbated by systemic inflammation or hypoxia following TBI [8].

Serum AST and ALT levels are routinely accessible clinical markers traditionally used in liver disease diagnosis. While ALT is primarily an indicator of liver dysfunction, AST levels can reflect cellular damage across multiple organs, including the brain, myocardium, kidney, and skeletal muscle [9]. The ratio between these enzymes, known as the De Ritis ratio (serum AST/ALT), was first described by De Ritis et al. as a method to distinguish between different types of hepatitis [10]. Recent research has expanded the clinical applications of the De Ritis ratio beyond hepatic conditions, establishing its value as a prognostic biomarker across various diseases and malignancies. Su et al., through a systematic review and meta-analysis, demonstrated its significance as a predictive indicator in urological cancer [11]. In cardiovascular medicine, Steininger et al. established the ratio’s role as a long-term independent mortality predictor following acute myocardial infarction, showing superior prognostic value compared to individual AST and ALT measurements [12]. Further cardiovascular applications were identified by Cao et al., who found that elevated De Ritis ratios at admission may indicate increased warfarin sensitivity in patients undergoing heart valve replacement surgery [13]. The ratio’s prognostic utility extends to neurological conditions, with increased values correlating with poor outcomes in acute ischemic stroke patients [14].

Given this growing evidence of the De Ritis ratio’s broad clinical utility, our study aims to investigate the relationship between initial De Ritis ratio values in emergency department presentations and clinical outcomes among patients with moderate-to-severe TBI.

## 2. Methods

### 2.1. Study Population

**Figure 1:**
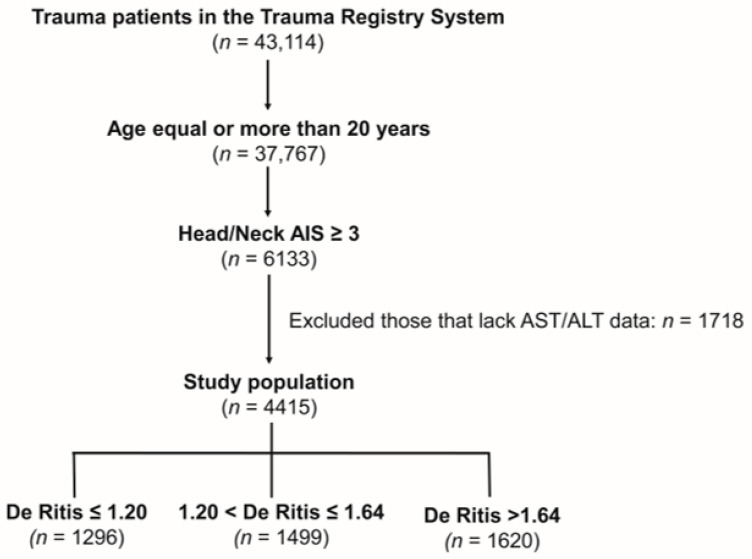
A flowchart showing how adult patients with a head and neck injury scale (AIS) score of 3 or more from traumatic brain injury were chosen and then put into three groups: De Ritis ratio ≤ 1.2, 1.2 < De Ritis ratio ≤ 1.64, and De Ritis ratio > 1.64. Those who did not have initial AST or ALT data upon hospital arrival were excluded from the study population.

This study retrospectively reviewed all trauma patients enrolled in the Trauma Registry System of the Chang Gung Memorial Hospital from 1 January 2009–31 December 2020 (Figure 1). From 43,114 patients, we included adult patients aged 20 years or older who suffered from moderate-to-severe TBI and who had a head and neck AIS ≥ 3. We excluded those patients (*n* = 1718) who did not have initial AST or ALT data upon hospital arrival. A total of 4415 patients with moderate-to-severe TBI were included in this study population. Patients were categorized into three groups: De Ritis ratio ≤ 1.2 (*n* = 1296), 1.2 < ratio ≤ 1.64 (*n* = 1499), and >1.64 (*n* = 1620). We used cut-off points of 1.2 and 1.64 to divide patients into three groups based on the De Ritis ratio in a study by Su et al. investigating 2248 adult patients with thoracoabdominal injuries [15]. Su et al. categorized the study population into three tertile groups based on the De Ritis ratio, designating a De Ritis ratio of 1.2 < ratio ≤ 1.64 as the reference group, under the premise that the De Ritis ratio of patients may fluctuate up or down following trauma incidents. In this study, the patients with a De Ritis ratio of 1.2 < ratio ≤ 1.64 were also used as a reference group.

Because the recommended cutoff values of 1.2 and 1.64 were based on the study performed on trauma patients with thoracoabdominal injuries [15], we performed further analysis on the patients who were stratified into tertiles based on the study population who had moderate-to-severe traumatic brain injury. The middle tertile as the reference category had been suggested when categorizing continuous scores to avoid assuming that either extreme is inherently beneficial or harmful [16,17,18]. Tertile cut-offs were determined from the entire cohort’s De Ritis ratio distribution. Patients with De Ritis ratio ≤ 1.25 comprised the lowest tertile, those with De Ritis ratio 1.26–1.70 the middle tertile, and those with De Ritis ratio > 1.70 the highest tertile. This stratification yielded 1473, 1478, and 1464 patients in the lowest, middle, and highest tertiles, respectively, with each group representing approximately one-third of the cohort.

The patients’ gender, age, serum AST and ALT levels, hemoglobin, platelets, prothrombin time (PT), activated partial thromboplastin time (aPTT), international normalized ratio (INR), comorbidities, Glasgow coma scale (GCS), injury severity score (ISS), length of stay (LOS) in the hospital, admission to an intensive care unit (ICU), and death in the hospital were all taken from a registered trauma database. The in-hospital mortality was defined as death from any cause occurring during the index hospitalization, and patients discharged alive were considered survivors for outcome analysis. Comorbidities included diabetes mellitus (DM), hypertension (HTN), coronary artery disease (CAD), congestive heart failure (CHF), cerebral vascular accident (CVA), and end-stage renal disease (ESRD). Patients with polytrauma were characterized as those exhibiting an AIS score of 3 or higher in two distinct body areas.

### 2.2. Statistical Analysis

The statistical analyses were conducted utilizing the Statistical Package for the Social Sciences software, version 23 for IBM Windows. The Kolmogorov–Smirnov test was employed to assess whether continuous variables were normally distributed. The Mann–Whitney U test was employed to assess non-normally distributed continuous data, which were presented as median with interquartile range (IQR) from Q1 to Q3. The categorical data were presented as counts and percentages and were analyzed using the two-sided Fisher’s exact test or Pearson’s chi-square test. The continuous data was presented as the mean ± standard deviation. To mitigate the bias from confounding variables related to baseline patient characteristics, a logistic regression model developed using Number Cruncher Statistical Systems (NCSS) 10 software (NCSS Statistical program, Kaysville, Utah) was implemented to compute the propensity scores, incorporating the following covariates that exhibited significant differences between groups: sex, age, co-morbidities, GCS, and ISS, utilizing a 1:1 greedy matching strategy with a caliper width of 0.2. A *p*-value below 0.05 was deemed statistically significant.

## 3. Results

### 3.1. Demographic Characteristics of the Study Population

As shown in Table 1, there were significantly more male patients, younger age, and higher ALT level (59.5 ± 81.2 vs. 44.2 ± 255.2 U/L) in the group with a De Ritis ratio ≤ 1.2 than in the reference group. No significant difference in the AST level (55.0 ± 74.5 vs. 62.2 ± 359.3 U/L) was found between these two groups of patients. The patients with a De Ritis ratio ≤ 1.2 had a significantly lower mortality rate (4.6% vs. 8.2%) and necessity for admission to the ICU (65.0% vs. 71.1%) than the patients in the group with a De Ritis ratio between 1.2 and 1.64. No significant difference in LOS in hospital was found between these two groups of patients.

The levels of AST and ALT were not significantly different between the patients in the group with a De Ritis ratio of 1.64 or higher and those in the reference group, albeit there seemed to be a higher AST (84.3 ± 253.7 vs. 62.2 ± 359.3 U/L) but lower ALT (34.5 ± 56.5 vs. 44.2 ± 255.2 U/L) level in the patients with a De Ritis ratio > 1.64 than in those in the reference group. Patients with a De Ritis ratio > 1.64, on the other hand, had higher PT (11.7 ± 4.8 vs. 11.1 ± 3.6 s), aPTT (28.2 ± 7.4 vs. 27.2 ± 5.9 s), and INR levels (1.08 ± 0.37 vs. 1.03 ± 0.26, *p* < 0.001), a lower rate of DM but a higher rate of ESRD, lower GCS, and higher ISS than the patients in the reference group. The patients who had a De Ritis ratio > 1.64 had a significantly higher rate of chest trauma than those patients with a De Ritis ratio of 1.2 < ratio ≤ 1.64 (13.1% vs. 9.2%, *p* < 0.001). The patients who had a De Ritis ratio ≤ 1.2 had a significantly lower rate of extremity trauma than those patients with a De Ritis ratio of 1.2 < ratio ≤ 1.64 (4.6% vs. 7.5%, *p* < 0.001). In addition, the patients who had a De Ritis ratio > 1.64 had a significantly higher rate of polytrauma than the reference group (20.7% vs. 16.5%, *p* < 0.001). Although the patients who had a De Ritis ratio ≤ 1.2 had a lower rate of polytrauma than the reference group (14.4% vs. 16.5%), the difference is not significant. The patients who had a De Ritis ratio > 1.64 were much more likely to die (17.6% vs. 8.2%), stay in the hospital longer (14.8 vs. 13.0 days), and need to be admitted to the ICU (77.5% vs. 71.1%) than the patients in the reference group.

### 3.2. Patient Outcomes of Propensity Score-Matched Cohorts

We made propensity score-matched patient cohorts for patients with De Ritis ratio ≤ 1.2 vs. the reference group (Table 2) and patients with De Ritis ratio > 1.64 vs. reference group (Table 3). For comparison, 1079 and 1185 patients were chosen from each group. In these matched patient cohorts, there was no significant difference in these baseline characteristics, including sex, age, comorbidities, GCS, and ISS. The laboratory data were excluded from matching, as they may indicate the reaction to trauma rather than solely the patient’s baseline characteristics.

When comparing the De Ritis ratio < 1.2 group to the reference group, these propensity score-matched cohorts showed that there was no significant difference in death rates (4.9% vs. 6.3%, odds ratio (OR) = 0.77, 95% confidence interval (CI) 0.53–1.11, *p* = 0.16) between the two. The mortality was 1.33-fold higher in the De Ritis ratio > 1.64 group (10.3% vs. 7.9%, OR = 1.33, 95% CI 1.01–1.77, *p* = 0.046) compared to the reference group. Both propensity score-matched cohorts showed no significant differences in hospital stays.

### 3.3. Patient Outcomes of Tertile Cut-Offs Derived from the Cohort’s De Ritis Ratio Distribution

Using tertile cut-offs derived from the cohort’s De Ritis ratio distribution (≤1.25, 1.26–1.70, >1.70), we observed similar patient outcomes that are in accordance with the prior literature-based grouping (Table 4). The only remarkable difference is that in the propensity score-matched patient cohort (Table 5), the “low De Ritis ratio” category (threshold ≤ 1.25) of tertile cut-off groups showed a clearer decrease in mortality outcomes than those patients with De Ritis ratio of 1.26–1.70 (OR 0.69; 95% CI 0.49–0.98; *p* = 0.036), whereas in the prior literature-based grouping, the “low De Ritis ratio” category (threshold ≤ 1.2) did not show a significant difference from those patients with De Ritis ratio of 1.2–1.64 (OR 0.77; 95% CI 0.53–1.11; *p* = 0.160) in mortality outcomes. The “high De Ritis ratio” category showed a significant difference from those patients with De Ritis ratio of 1.2–1.64 (Table 6), a similar result as that found in the prior literature-based grouping. The tertile-based analysis revealed a more graded risk pattern across low, mid, and high De Ritis ratio groups than those analyses from prior literature-based groupings.

## 4. Discussion

AST is a crucial enzyme in glutamate metabolism. Its primary function involves catalyzing the transfer of amino groups from aspartate to 2-oxoglutarate, resulting in the formation of oxaloacetate and glutamate. This enzymatic process plays a vital role in maintaining glutamate homeostasis in the brain. In acute ischemic stroke, research has demonstrated that lower AST levels correlate with elevated blood glutamate levels, leading to poorer functional outcomes [19]. Studies have shown significantly higher glutamate concentrations in both plasma and cerebrospinal fluid of stroke patients, correlating with larger cerebral infarction sizes. The excessive glutamate activity triggers excitotoxicity, promoting cell death and brain injury, ultimately resulting in early neurological deficit progression [20,21].

Further research by Muscari et al. revealed that AST is the only liver enzyme directly influenced by brain infarction volume. Their hypothesis suggests that AST production serves as a neuroprotective mechanism, specifically aimed at neutralizing glutamate’s neurotoxic effects in stroke patients [22]. This protective role is supported by findings from Campos et al., who demonstrated that elevated serum AST levels correlate with improved outcomes in acute ischemic stroke patients [19,23]. Guerriero et al. described at the molecular level in TBI how membrane swelling and potential rupture lead to glutamate release into the extracellular space, perpetuating neuronal injury [24]. In response to brain injury detection, serum AST production is activated to facilitate glutamate removal from brain tissue through blood glutamate degradation. Consequently, patients with more severe TBI maintain higher proliferative states of serum AST compared to serum ALT, resulting in an elevated De Ritis ratio. Notably, in this study, the levels of AST and ALT were not significantly different between the patients in the group with a De Ritis ratio of 1.64 or higher and those in the reference group. Although there was a higher AST (84.3 ± 253.7 vs. 62.2 ± 359.3 U/L) but lower ALT (34.5 ± 56.5 vs. 44.2 ± 255.2 U/L) level in the patients with a De Ritis ratio > 1.64 than in those in the reference group, the difference was not significant. However, the De Ritis ratio, calculated by dividing the value of AST by the value of ALT, indicates the associated mortality risk of the patients with moderate-to-severe TBI.

In this study, the older individuals tend to have higher De Ritis ratios, which is in accordance with those reported in literature [25,26]. Therefore, matching on age would attenuate the influence of age on the impact of the De Ritis ratio on the outcome assessment. However, regarding the optimal cut-off point, the De Ritis ratio lacks a universal consensus. This threshold varies considerably across different disease states and clinical scenarios. In cardiovascular conditions, a De Ritis ratio exceeding 1.2 has been identified as an indicator of increased risk for acute myocardial infarction [12]. Similarly, for patients undergoing heart valve replacement surgery, a ratio above 1.19 at admission suggests increased sensitivity to warfarin therapy [13]. In neurological conditions, specifically in first-ever acute ischemic stroke patients, a De Ritis ratio greater than 1.53 at admission has been significantly associated with poorer outcomes [14]. The ratio’s prognostic value extends to oncological conditions as well. In metastatic renal cell carcinoma patients receiving first-line systemic tyrosine kinase inhibitor therapy, a De Ritis ratio ≥ 1.2 correlates with worse cancer-specific survival and overall survival outcomes [27]. Similarly, for surgically treated localized clear-cell renal cell carcinoma patients, a ratio ≥ 1.5 indicates poorer postoperative survival [28]. More recent clinical applications include COVID-19 patients, where a De Ritis ratio ≥ 1.63 on admission shows significant association with in-hospital mortality [29]. In hematological conditions, particularly in adult secondary hemophagocytic lymphohistiocytosis, a ratio exceeding 1.79 serves as a reliable predictor for overall survival [30]. In this study, we used cut-off points of 1.2 and 1.64 to divide patients into three groups based on the De Ritis ratio in a previous study of 2248 adult patients with thoracoabdominal injuries, which included some patients with direct liver trauma [15]. Although the patient population in this study cohort also comprised some patients with thoracoabdominal injuries, the thresholds can be different for different diseases. The odds of risk may be varied based on the selection of cut-off points of the De Ritis ratio for the reference group; however, the association of higher mortality risk in those with a high De Ritis ratio seemed to be retained, seeing there was a trend for a higher AST but lower ALT level of the patients with a higher De Ritis ratio.

In this study, the tertile-based analysis revealed a more graded risk pattern across low, mid, and high De Ritis ratio groups than those analyses from prior literature-based groupings, which are based on the study on trauma patients with thoracoabdominal injury. Although these findings underscore that a data-driven tertile grouping of the De Ritis ratio can meaningfully alter its prognostic interpretation in trauma ICU patients, a worse outcome was found in each cohort for those with an elevated De Ritis ratio, which predominantly flags patients with high injury-induced inflammatory burden. This echoes findings by others that elevated CRP-based indices often reflect underlying injury complexity and correlate with poor outcomes [31]. Furthermore, the tertile-based De Ritis ratio grouping appears to better capture the gradation of risk in critically injured patients, thereby offering potentially more reliable risk stratification for guiding management in the patient cohort [32].

Our study faced several important limitations that warrant consideration. As data collection was confined to a single medical center, the findings may not fully represent the general population, and the retrospective study design inherently introduces potential selection bias. A key methodological constraint was our exclusive focus on the relationship between emergency department liver enzyme ratios and TBI outcomes, without accounting for subsequent complications during hospitalization that could significantly impact patient morbidity and mortality, thus affecting the prognostic value of the initial De Ritis ratio. Additionally, while the OR of 1.33 is statistically significant, its clinical magnitude is modest, suggesting that the De Ritis ratio is unlikely to be the primary predictor of patient outcomes. Furthermore, the predictive power of the De Ritis ratio may be influenced by various confounding factors affecting initial serum AST and ALT levels, including multiple trauma with concurrent organ injuries, pre-existing liver conditions, pre-existing medical conditions, previous medication effects, toxin exposure, and systemic inflammatory responses, all of which could impact the accuracy of this prognostic indicator. In this study, deranged laboratory findings such as hemoglobin and glycemic levels were found in patients with higher De Ritis ratios. Notably, low serum hemoglobin, high blood glucose, high base excess, low mean arterial pressure, and low PaO2/FiO2 ratio are all associated with increased mortality in patients admitted to the ICU following severe TBI [33]. However, the laboratory data were excluded from matching, as they may indicate the reaction to trauma rather than solely the patient’s baseline characteristics, which may result in bias in the data analysis. Additionally, the comparison of separate propensity score-matched patient cohorts against a reference group may yield different analytical conclusions compared to those obtained by three-way matching [34]. Given that ICU care has influenced the management of a wide range of TBI in many clinical settings, additional research on patient outcomes and quality of life metrics is required to determine their efficacy [35].

## 5. Conclusions

The De Ritis ratio represents a promising prognostic marker for moderate-to-severe TBI patients. Our study demonstrates that a higher initial De Ritis ratio (>1.64) significantly correlates with increased mortality rates compared to reference groups, even after adjusting for confounding factors through propensity score matching. This readily available laboratory parameter could serve as a simple yet effective tool for early risk assessment in TBI patients, potentially aiding clinicians in treatment planning and patient care optimization.

## Figures and Tables

**Table 1:** Characteristics of patients, injuries, and laboratory data of the studied population.

Variables	De Ritis Ratio			
	≤1.2 *n* = 1296	1.2 < Ratio ≤ 1.64 *n* = 1499	>1.64 *n* = 1620	*p*
Male, *n* (%)	911 (70.3) *	889 (59.3)	956 (59.0)	<0.001
Age, years	54.9 ± 18.1 *	58.1 ± 19.1	59.1 ± 19.6	<0.001
AST, (U/L)	55.0 ± 74.5	62.2 ± 359.3	84.3 ± 253.7	0.006
OR (95% CI) ^†^	1.00 (1.00–1.00)	-	1.01 (1.00–1.01)	
ALT, (U/L)	59.5 ± 81.2 *	44.2 ± 255.2	34.5 ± 56.5	<0.001
OR (95% CI) ^†^	1.02 (1.01–1.03)	-	0.99 (0.98–1.00)	
Hemoglobin, (g/dL)	13.5 ± 7.5	13.1 ± 2.0	13.0 ± 2.2	0.008
Platelet, (1000/μL)	219.7 ± 64.8	225.3 ± 73.1	222.9 ± 73.1	0.120
PT, (s)	10.9 ± 2.4	11.1 ± 3.6	11.7 ± 4.8 ^†^	<0.001
aPTT, (s)	26.9 ± 5.1	27.2 ± 5.9	28.2 ± 7.4 ^†^	<0.001
INR	1.03 ± 0.24	1.03 ± 0.26	1.08 ± 0.37 ^†^	<0.001
Comorbidities				
CVA, *n* (%)	61 (4.7)	77 (5.1)	91 (5.6)	0.542
HTN, *n* (%)	474 (36.6)	552 (36.8)	552 (34.1)	0.210
CAD, *n* (%)	73 (5.6)	104 (6.9)	112 (6.9)	0.286
CHF, *n* (%)	7 (0.5)	10 (0.7)	14 (0.9)	0.570
DM, *n* (%)	288 (22.2)	300 (20.0)	268 (16.5) ^†^	<0.001
ESRD, *n* (%)	28 (2.2)	33 (2.2)	59 (3.6) ^†^	0.016
GCS, median (IQR)	15 (12–15)	15 (11–15)	13 (7–15) ^†^	<0.001
AIS ≥ 3				
Face, *n* (%)	10 (0.8)	9 (0.6)	8 (0.5)	0.632
Chest, *n* (%)	123 (9.5)	138 (9.2)	212 (13.1) ^†^	0.001
Abdomen, *n* (%)	29 (2.2)	22 (1.5)	34 (2.1)	0.274
Extremities, *n* (%)	60 (4.6) *	112 (7.5)	143 (8.8)	<0.001
External, *n* (%)	1 (0.1)	0 (0.0)	0 (0.0)	0.300
Polytrauma (AIS ≥ 3 in two regions), *n* (%)	187 (14.4)	248 (16.5)	335 (20.7) ^†^	<0.001
ISS, median (IQR)	16 (14–21)	16 (16–24)	20 (16–25) ^†^	<0.001
Mortality, *n* (%)	60 (4.6) *	123 (8.2)	285 (17.6) ^†^	<0.001
LOS in hospital (days)	13.0 ± 14.0	13.0 ± 12.7	14.8 ± 15.1 ^†^	<0.001
Admission into ICU, *n* (%)	842 (65.0) *	1066 (71.1)	1256 (77.5) ^†^	<0.001

AIS = Abbreviated Injury Scale; CAD = coronary artery disease; CHF = congestive heart failure; CI = confidence interval; CVA = cerebral vascular accident; DM = diabetes mellitus; ESRD = end-stage renal disease; GCS = Glasgow Coma Scale; HTN = hypertension; IQR = interquartile range; ISS = injury severity score; OR= odds ratio. * indicates a significant difference in the patients with a De Ritis ratio ≤ 1.2 in comparison with those reference patients with a De Ritis ratio of 1.2 < ratio ≤ 1.64. ^†^ indicates a significant difference in the patients with a De Ritis ratio > 1.64 in comparison with those reference patients with a De Ritis ratio of 1.2 < ratio ≤ 1.64. Comparisons across the three De Ritis ratio groups were made using one-way ANOVA for normally distributed continuous variables, the Mann–Whitney U test for non-normally distributed continuous data, and chi-square test for categorical variables.

**Table 2:** Propensity score-matched patient cohort of the patients with a De Ritis ratio ≤ 1.2 vs. those in the reference group with a De Ritis ratio of 1.2 < ratio ≤ 1.64.

	De Ritis Ratio				
	≤1.2 *n* = 1079	1.2 < Ratio ≤ 1.64 *n* = 1079	OR (95%CI)	*p*	SD
Male, *n* (%)	747 (69.2)	747 (69.2)	1.00 (0.83–1.20)	1.000	0.00%
Age, years	55.3 ± 18.2	55.6 ±18.6	-	0.715	−1.57%
CVA, *n* (%)	40 (3.7)	40 (3.7)	1.00 (0.64–1.56)	1.000	0.00%
HTN, *n* (%)	367 (34.0)	367 (34.0)	1.00 (0.84–1.20)	1.000	0.00%
CAD, *n* (%)	50 (4.6)	50 (4.6)	1.00 (0.67–1.49)	1.000	0.00%
CHF, *n* (%)	1 (0.1)	1 (0.1)	1.00 (0.06–16.01)	1.000	0.00%
DM, *n* (%)	213 (19.7)	213 (19.7)	1.00 (0.81–1.24)	1.000	0.00%
ESRD, *n* (%)	12 (1.1)	12 (1.1)	1.00 (0.45–2.24)	1.000	0.00%
GCS, median (IQR)	15 (11–15)	15 (12–15)	-	0.774	−1.24%
ISS, median (IQR)	16 (16–22)	16 (16–22)	-	0.734	1.46%
Mortality, *n* (%)	53 (4.9)	68 (6.3)	0.77 (0.53–1.11)	0.160	-
Hospital stay, days	13.1 ± 14.2	13.0 ± 12.5	-	0.923	-

CAD = coronary artery disease; CHF = congestive heart failure; CI = confidence interval; CVA = cerebral vascular accident; DM = diabetes mellitus; ESRD = end-stage renal disease; HTN = hypertension; IQR = interquartile range; ISS = injury severity score; OR = odds ratio; SD = standardized differences. In the propensity score-matched cohort (De Ritis ratio ≤ 1.2 vs. reference 1.2–1.64), categorical variables were compared by chi-square/Fisher’s exact tests and continuous variables by the Mann–Whitney U test. A logistic regression model was used to calculate the odds ratio (OR) and 95% confidence interval for mortality between the two matched groups.

**Table 3:** Propensity score-matched patient cohort of the patients with a De Ritis ratio > 1.64 vs. those in the reference group with a De Ritis ratio of 1.2 < ratio ≤ 1.64.

	De Ritis Ratio				
	>1.64 *n* = 1185	1.2 < Ratio ≤ 1.64 *n* = 1185	OR (95%CI)	*p*	SD
Male, *n* (%)	710 (59.9)	710 (59.9)	1.00 (0.85–1.18)	1.000	0.00%
Age, years	57.6 ± 19.3	57.8 ± 18.8	-	0.816	−0.96%
CVA, *n* (%)	46 (3.9)	46 (3.9)	1.00 (0.66–1.52)	1.000	0.00%
HTN, *n* (%)	392 (33.1)	392 (33.1)	1.00 (0.84–1.19)	1.000	0.00%
CAD, *n* (%)	56 (4.7)	56 (4.7)	1.00 (0.68–1.46)	1.000	0.00%
CHF, *n* (%)	1 (0.1)	1 (0.1)	1.00 (0.06–16.01)	1.000	0.00%
DM, *n* (%)	193 (16.3)	193 (16.3)	1.00 (0.80–1.24)	1.000	0.00%
ESRD, *n* (%)	11 (0.9)	11 (0.9)	1.00 (0.43–2.32)	1.000	0.00%
GCS, median (IQR)	15 (9–15)	15 (10–15)	-	0.668	−1.76%
ISS, median (IQR)	17 (6–24)	17 (16–25)	-	0.991	0.05%
Mortality, *n* (%)	122 (10.3)	97 (7.9)	1.33 (1.01–1.77)	0.046 ^†^	-
Hospital stay, days	14.2 ± 14.0	13.7 ± 13.2	-	0.425	-

CAD = coronary artery disease; CHF = congestive heart failure; CI = confidence interval; CVA = cerebral vascular accident; DM = diabetes mellitus; ESRD = end-stage renal disease; HTN = hypertension; IQR = interquartile range; ISS = injury severity score; OR = odds ratio; SD = standardized differences. In the propensity score-matched cohort (De Ritis ratio > 1.64 vs. reference), categorical data were compared by chi-square or Fisher’s exact tests and continuous data by Mann–Whitney U test. Multivariate logistic regression provided the OR (95% CI) for in-hospital mortality in the high De Ritis group relative to the reference group. † indicates a significant difference in the patients with a De Ritis ratio > 1.64 in comparison with those reference patients with a De Ritis ratio of 1.2 < ratio ≤ 1.64.

**Table 4:** Characteristics of patients, injuries, and laboratory data based on tertile of the studied population.

Variables	De Ritis Ratio			
	≤1.25 *n* = 1473	1.26 < Ratio ≤ 1.70 *n* = 1478	>1.70 *n* = 1464	*p*
Male, *n* (%)	1022 (69.4) *	855 (57.8)	879 (60.0)	<0.001
Age, years	55.1 ± 18.2 *	58.4 ± 19.1	59.1 ± 19.7	<0.001
AST, (U/L)	54.4 ± 73.6	63.6 ± 362.2	86.7 ± 265.3 ^†^	0.003
OR (95% CI) ^†^	1.00 (0.99–1.00)	-	1.01 (1.00–1.01)	
ALT, (U/L)	57.3 ± 78.7 *	43.8 ± 256.9	34.2 ± 57.0	<0.001
OR (95% CI) ^†^	1.02 (1.01–1.03)	-	0.99 (0.98–1.00)	
Hemoglobin, (g/dL)	13.4 ± 7.1	13.1 ± 2.0	13.0 ± 2.2	0.032
Platelet, (1000/μL)	220.0 ± 64.9	224.9 ± 74.2	223.4 ± 73.0	0.150
PT, (s)	10.9 ± 2.3	11.1 ± 3.7	11.8 ± 5.1 ^†^	<0.001
aPTT, (s)	27.0 ± 5.0	27.2 ± 5.9	28.3 ± 7.7 ^†^	<0.001
INR	1.0 ± 0.2	1.0 ± 0.3	1.1 ± 0.4 ^†^	<0.001
Comorbidities				
CVA, *n* (%)	71 (4.8)	72 (4.9)	86 (5.9)	0.348
HTN, *n* (%)	536 (36.4)	546 (36.9)	496 (33.9)	0.182
CAD, *n* (%)	87 (5.9)	100 (6.8)	102 (7.0)	0.466
CHF, *n* (%)	8 (0.5)	10 (0.7)	13 (0.9)	0.529
DM, *n* (%)	325 (22.1)	291 (19.7)	240 (16.4) ^†^	<0.001
ESRD, *n* (%)	35 (2.4)	30 (2.0)	55 (3.8) ^†^	0.010
GCS, median (IQR)	15 (12–15) *	15 (11–15)	13 (7–15) ^†^	<0.001
AIS ≥ 3				
Face, *n* (%)	11 (0.7)	7 (0.5)	27 (0.6)	0.636
Chest, *n* (%)	135 (9.2)	150 (10.1)	188 (12.8) ^†^	0.004
Abdomen, *n* (%)	30 (2.0)	22 (1.5)	33 (2.3)	0.297
Extremities, *n* (%)	68 (4.6) *	113 (7.6)	134 (9.2)	<0.001
External, *n* (%)	1 (0.1)	0 (0.0)	0 (0.0)	0.368
Polytrauma (AIS ≥ 3 in two regions), *n* (%)	207 (14.1) *	257 (17.4)	306 (20.9) ^†^	<0.001
ISS, median (IQR)	16 (14–21) *	16 (16–24)	20 (16–25) ^†^	<0.001
Mortality, *n* (%)	74 (5.0) *	127 (8.6)	267 (18.2) ^†^	<0.001
LOS in hospital (days)	13.0 ± 13.8	13.1 ± 12.8	14.9 ± 15.2 ^†^	<0.001
Admission into ICU, *n* (%)	969 (65.8) *	1055 (71.4)	1140 (77.9) ^†^	<0.001

AIS = Abbreviated Injury Scale; CAD = coronary artery disease; CHF = congestive heart failure; CI = confidence interval; CVA = cerebral vascular accident; DM = diabetes mellitus; ESRD = end-stage renal disease; GCS = Glasgow Coma Scale; HTN = hypertension; IQR = interquartile range; ISS = injury severity score; OR= odds ratio. * indicates a significant difference in the patients with a De Ritis ratio ≤ 1.25 in comparison with those reference patients with a De Ritis ratio of 1.25 < ratio ≤ 1.70. ^†^ indicates a significant difference in the patients with a De Ritis ratio > 1.70 in comparison with those reference patients with a De Ritis ratio of 1.25 < ratio ≤ 1.70. Comparisons across the three De Ritis ratio groups were made using one-way ANOVA for normally distributed continuous variables, the Mann–Whitney U test for non-normally distributed continuous data, and chi-square test for categorical variables.

**Table 5:** Propensity score-matched patient cohort of the patients with a De Ritis ratio ≤1.25 vs. those in the reference group with a De Ritis ratio of 1.26 < ratio ≤ 1.70.

	De Ritis Ratio				
	≤1.25 *n* = 1139	1.26 < Ratio ≤ 1.70 *n* = 1139	OR (95%CI)	*p*	SD
Male, *n* (%)	751 (65.9)	751 (65.9)	1.00 (0.84–1.19)	1.000	0.00%
Age, years	56.0 ± 18.1	56.4 ± 18.7	-	0.559	−2.45%
CVA, *n* (%)	37 (3.2)	37 (3.2)	1.00 (0.63–1.59)	1.000	0.00%
HTN, *n* (%)	401 (35.2)	401 (35.2)	1.00 (0.84–1.19)	1.000	0.00%
CAD, *n* (%)	47 (4.1)	47 (4.1)	1.00 (0.66–1.51)	1.000	0.00%
CHF, *n* (%)	1 (0.1)	1 (0.1)	1.00 (0.06–16.01)	1.000	0.00%
DM, *n* (%)	222 (19.5)	222 (19.5)	1.00 (0.81–1.23)	1.000	0.00%
ESRD, *n* (%)	12 (1.1)	12 (1.1)	1.00 (0.45–2.24)	1.000	0.00%
GCS, median (IQR)	15 (11–15)	15 (11–15)	-	0.904	0.51%
ISS, median (IQR)	16 (16–22)	16 (16–22)	-	0.628	2.03%
Mortality, *n* (%)	58 (5.1)	82 (7.2)	0.69 (0.49–0.98)	0.036 *	-
Hospital stay, days	13.3 ± 14.3	13.1 ± 12.5	-	0.704	-

CAD = coronary artery disease; CHF = congestive heart failure; CI = confidence interval; CVA = cerebral vascular accident; DM = diabetes mellitus; ESRD = end-stage renal disease; HTN = hypertension; IQR = interquartile range; ISS = injury severity score; OR = odds ratio; SD = standardized differences. For the propensity score-matched De Ritis ratio groups (low De Ritis ratio group vs. middle-tertile reference group), categorical variables were compared with chi-square/Fisher’s exact tests and continuous variables with the Mann–Whitney U test. Logistic regression was used to estimate ORs (95% CI) for mortality comparing the low De Ritis ratio group to the middle tertile reference group. * indicates a significant difference in the patients with a De Ritis ratio ≤1.25 in comparison with those reference patients with a De Ritis ratio of 1.25 < ratio ≤ 1.70.

**Table 6:** Propensity score-matched patient cohort of the patients with a De Ritis ratio > 1.64 vs. those in the reference group with a De Ritis ratio of 1.26 < ratio ≤ 1.70.

	De Ritis Ratio				
	>1.70 *n* = 1131	1.26 < Ratio ≤ 1.70 *n* = 1131	OR (95%CI)	*p*	SD
Male, *n* (%)	679 (60.0)	679 (60.0)	1.00 (0.85–1.18)	1.000	0.00%
Age, years	57.5 ± 19.8	57.8 ± 19.0	-	0.725	−1.48%
CVA, *n* (%)	43 (3.8)	43 (3.8)	1.00 (0.65–1.54)	1.000	0.00%
HTN, *n* (%)	361 (31.9)	361 (31.9)	1.00 (0.84–1.19)	1.000	0.00%
CAD, *n* (%)	50 (4.4)	50 (4.4)	1.00 (0.67–1.49)	1.000	0.00%
CHF, *n* (%)	0 (0.0)	0 (0.0)	-	-	-
DM, *n* (%)	180 (15.9)	180 (15.9)	1.00 (0.80–1.25)	1.000	0.00%
ESRD, *n* (%)	11 (1.0)	11 (1.0)	1.00 (0.43–2.32)	1.000	0.00%
GCS, median (IQR)	14 (9–15)	14 (9–15)	-	0.812	−1.00%
ISS, median (IQR)	17 (16–25)	17 (16–25)	-	0.461	3.10%
Mortality, *n* (%)	130 (11.5)	101 (8.9)	1.32 (1.01–1.74)	0.044 ^†^	-
Hospital stay, days	14.1 ± 13.7	13.7 ± 13.3	-	0.476	-

CAD = coronary artery disease; CHF = congestive heart failure; CI = confidence interval; CVA = cerebral vascular accident; DM = diabetes mellitus; ESRD = end-stage renal disease; HTN = hypertension; IQR = interquartile range; ISS = injury severity score; OR = odds ratio; SD = standardized differences. For the propensity score-matched De Ritis ratio groups (high De Ritis ratio vs. middle tertile), categorical comparisons used chi-square or Fisher’s exact tests and continuous comparisons used Mann–Whitney U tests. A logistic regression analysis provided the OR (95% CI) for mortality in the high De Ritis ratio group versus the middle tertile group. ^†^ indicates a significant difference in the patients with a De Ritis ratio > 1.70 in comparison with those reference patients with a De Ritis ratio of 1.25 < ratio ≤ 1.70.

## Data Availability

The de-identificated data could be provided via corresponding author for academic research purpose.
